# NC-6004 Phase I study in combination with gemcitabine for advanced solid tumors and population PK/PD analysis

**DOI:** 10.1007/s00280-017-3254-4

**Published:** 2017-02-21

**Authors:** Toshihiko Doi, Tetsuya Hamaguchi, Kohei Shitara, Satoru Iwasa, Yasuhiro Shimada, Mitsunori Harada, Kenichiro Naito, Naoto Hayashi, Atsuhiro Masada, Atsushi Ohtsu

**Affiliations:** 1 Exploratory Oncology Research and Clinical Trial Center, National Cancer Center Hospital East, Chiba, Japan; 2 Gastrointestinal Oncology Division, National Cancer Center Hospital, Tokyo, Japan; 3 NanoCarrier Co. Ltd., Onoya Kyobashi Building, 1-4-10 Kyobashi, Chuo-ku, Tokyo, 104-0031 Japan

**Keywords:** Acute kidney injury, Cisplatin, Micelles, Population PK/PD analysis, NC-6004

## Abstract

**Objectives:**

This study was an open-label phase I study to confirm the safety and tolerability of NC-6004 in combination with gemcitabine in Japanese patients with advanced solid tumors and to assess the PK effects of NC-6004 monotherapy.

**Methods:**

This phase I study used a 3 + 3 design to determine the maximum tolerated dose (MTD) and recommended dose of NC-6004 combined with gemcitabine. Safety and pharmacokinetics were assessed. The administration of NC-6004 alone was started at 60 mg/m^2^ every treatment cycle (21 days per cycle). From the second through eighth cycles, patients received NC-6004 in combination with 1000 mg/m^2^ of gemcitabine that was administered on day 1 and day 8 of each cycle, except for the first treatment cycle.

**Results:**

Twelve patients with advanced solid tumors received 60 or 90 mg/m^2^ NC-6004. Both MTD and RD were determined to be 90 mg/m^2^. The most common drug-related adverse events were neutrophil decrease (66.7%) and white blood cell count decrease (41.7%). Population pharmacokinetic (PK) analysis revealed that NC-6004 PK profile in Japanese study was not significantly different from that in a previous Caucasian study.

**Conclusions:**

Both MTD and RD of NC-6004 were determined to be 90 mg/m^2^. The pharmacodynamic (PD) model well explained the time course of estimated glomerular filtration rate (eGFR) and amplitude of decrease in eGFR. The decrease in eGFR appeared to reach saturation at >100 mg/m^2^ with NC-6004. Estimated probability of acute kidney injury on this PK/PD simulation was 30% with NC-6004 and 70% with cisplatin, which may better explain the renal toxicity profile.

## Introduction

NC-6004 is a novel micellar nanoparticle product of cisplatin, approximately 30 nm in diameter, obtained by cross-linking with a polyethylene glycol–poly glutamic acid block copolymer. Early basic research has indicated that when compared with conventional cisplatin, encapsulating cisplatin in the micellar nanoparticle can selectively improve accumulation at the tumor site. Indeed, NC-6004 progressively breaks down in the presence of chloride to release cisplatin slowly, and this slow release helps achieve longer systemic exposure that contributes to continuous and potentially improved antitumor effects. Cytotoxicity from NC-6004 is expected based on the formation of cellular DNA adducts with the released cisplatin.

Preliminary nonclinical studies have indicated several beneficial characteristics of NC-6004: (1) preferential distribution to tumors, (2) significantly lower toxicity compared with cisplatin at equivalent doses, and (3) increased antitumor activity [1]. The enhanced permeability and retention associated with polymeric micelles mean that the formulation benefited from extended blood circulation and selective and higher accumulation at the tumor site [2]. These clinical characteristics of NC-6004 have been demonstrated in both a phase I study where NC-6004 was used in monotherapy for patients with solid tumors in the UK (the NC-6004-001 study) and a phase I/II study where NC-6004 was used in combination with gemcitabine in patients with pancreatic cancer in Taiwan and Singapore (the NC-6004-002 study).

The present study was designed with two aims: (1) to assess the tolerability of NC-6004 in combination with gemcitabine in Japanese patients with solid tumors and (2) to obtain pharmacokinetic (PK) and pharmacodynamic (PD) data for NC-6004 monotherapy in Japanese patients.

## Materials and methods

### Study design

This study was an open-label phase I study to confirm the safety and tolerability of NC-6004 in combination with gemcitabine in Japanese patients with advanced solid tumors and to assess the PK effects of NC-6004 monotherapy. The study schedule comprised a screening phase, an NC-6004-alone phase, an NC-6004-plus-gemcitabine combination phase, and a follow-up phase. The study was conducted according to the provisions of the Declaration of Helsinki and the Guidelines for Good Clinical Practice, and was approved by the relevant institutional review board. Written informed consent was obtained from all patients before inclusion.

### Patient selection

Japanese patients with treatment-refractory solid tumors were recruited. Inclusion criteria were as follows: (1) age between 20 and 75 years, (2) histologically or cytologically confirmed cancer, (3) evaluable tumor lesions according to the Response Evaluation Criteria in Solid Tumors guideline (Version 1.1), (4) Eastern Cooperative Oncology Group performance status 0 to 2, and (5) adequate bone marrow reserve at screening. The exclusion criteria were as follows: (1) known hypersensitivity to platinum compounds or gemcitabine; (2) previous therapy with more than two different platinum-based regimens, or a regimen with a cumulative dose exceeding 480 mg/m^2^ for cisplatin, 1040 mg/m^2^ for oxaliplatin, or 42 mg/mL/min for carboplatin (cumulative area under the curve); (3) previous chemotherapy or radiotherapy within 28 days before the study treatment; (4) a history of symptomatic pulmonary fibrosis or interstitial pneumonia, with obvious evidence on a plain X-ray of the chest; (5) previous chest radiotherapy; (6) diagnosed chronic kidney disease defined as an estimated glomerular filtration rate (eGFR) <60 mL/min/1.73 m^2^; and (7) greater than grade 2 auditory toxicity by pure tone audiometry or greater than grade 2 neurotoxicity.

### Dosing rationale

Although the MTD was not determined during the NC-6004-001 phase I study in the UK, the RD was suggested to be close to 90 mg/m^2^ for monotherapy. The RD of NC-6004 given with gemcitabine has also been estimated to be 90 mg/m^2^ based on the phase I part of the NC-6004-002 study in Asia. From those results, the RD of NC-6004 in the Japanese patient population has been estimated at 90 mg/m^2^; however, in this study we initiated dosing at 60 mg/m^2^, and NC-6004 was mixed in 250 mL of 5% dextrose solution and given intravenously for over 60 min daily for 21 days. Oral dexamethasone; normal saline solution, including KCl and MgSO4; diphenhydramine hydrochloride; ranitidine; and mannitol were also provided as a prophylactic treatment for hypersensitivity before and after each treatment cycle. Treatment could be continued until eight cycles over 24 weeks, with the patient observed until 28 days after the eighth cycle or until discontinuation of the study treatment.

### Protocol: determination of the MTD and RD

The administration of NC-6004 alone was started at 60 mg/m^2^ every treatment cycle (21 days per cycle). From the second through eighth cycles, patients received NC-6004 in combination with 1,000 mg/m^2^ of gemcitabine that was administered on day 1 and day 8 of each cycle, except for the first treatment cycle.

Three patients were expected to be enrolled in the NC-6004 60 mg/m^2^ cohort. When a dose-limiting toxicity (DLT) event was observed, another three patients would be added to assess safety. A maximum of nine patients could be enrolled, as necessary, to obtain further safety information in this cohort. If two of the six or nine patients reached a DLT event at 60 mg/m^2^, that was considered the MTD. After evaluating safety for all patients, including at least two cycles of the study treatment, the first patient in the next 90 mg/m^2^ cohort was enrolled. Subsequent dose escalations were planned to doses of 120 or 150 mg/m^2^ in the same manner. The MTD was defined as the dose at which two of the six or nine patients experienced DLT. The RD was defined as the dose one level lower than the MTD; however, in all cases, the MTD or RD was only confirmed after discussion between the sponsor and the medical expert.

DLT was defined as follows: (1) grade 4 hematologic toxicity; (2) grade 3 thrombocytopenia with bleeding; (3) grade 3 or greater neutropenia with fever over 38.5 °C or with grade 2 or greater diarrhea; (4) grade 3 or greater neutropenia without fever for at least five days; (5) grade 3 or greater non-hematologic toxicity (except alopecia, treatable nausea or emesis, and biochemistry abnormalities without specific symptoms); (6) treatment delay of greater than two weeks before the start of the next treatment cycle (NC-6004 or gemcitabine) due to unresolved toxicity; (7) grade 3 or greater hypersensitivity reaction; (8) an eGFR toxicity of 30–59 mL/min/1.73 m^2^ delaying dosing by more than 14 days; or (9) an eGFR <30 mL/min/1.73 m^2^, or apparent renal failure identified by another index.

### Statistical analysis

Data were not tested statistically because this phase I study focused on safety and tolerability, estimation of the RD, and collection of PK data, except in the case of the population analysis.

### Population analysis

All population analyses were performed by NONMEM ver. 7.3.0, using the FOCE INTER estimation method. A difference of 7.879 in the value of the objective function (ΔOFV) with one degree of freedom was defined as being statistically significant (*P* < 0.005). A proportional model was selected for the residual error and inter-individual variability for the stochastic model.

### Population PK analysis

Blood samples for PK analysis were taken immediately before dosing, just after dosing, and at 1, 3, 6, 24, 48, 72, 168, 336, and 504 h after dosing. A population PK analysis was then performed using data obtained in this study that were merged with those from the phase I study performed by NanoCarrier Co., Ltd. in the UK (NC-6004-001 study).

The plasma total platinum (Pt) concentration was measured by the inductively coupled plasma (ICP) method in this study. In the previous UK study, plasma total Pt concentration was measured by both ICP and atomic absorption spectroscopy (AA) methods. Outlier data were not excluded from the analysis. In this PK analysis, the ratio of values between ICP and AA had an intra-individual distribution and the AA value was converted to an ICP value.

Two compartments with a first-order elimination model were selected for PK structural model. A proportional model was selected for the residual error and inter-individual variability as the stochastic model.

As the possible/available covariates, baselines of body weight and eGFR and ethnic difference were selected. Clearance and volume of distribution might be influenced by these covariates.

First, all possible combinations of covariates and PK parameters were included in the base model, and non-significant covariates were then excluded. The models including all significant covariates were tested for each covariate, and the significant covariates remained in the final model.

### Population PK/PD analysis

A population PK/PD model for eGFR was built with an empirical indirect model of Model I [3], using the following equation for the eGFR time course:

$$\frac{d~eGFR}{dt}={{k}_{in}}\times \left( 1-\frac{{{I}_{max}}\times {{C}_{f}}}{I{{C}_{50}}+{{C}_{f}}} \right)-{{k}_{out}}\times eGFR,$$

where GFR_0_ is the baseline GFR, *I*_max_ is the maximum inhibition (0 < *I*_max_ < 1), k_in_ is eGFR_0_ × *K*_out_, IC_50_ is the concentration to achieve 50% inhibition, and *C*_f_ is the plasma-free Pt concentration.

**Fig. 1:**
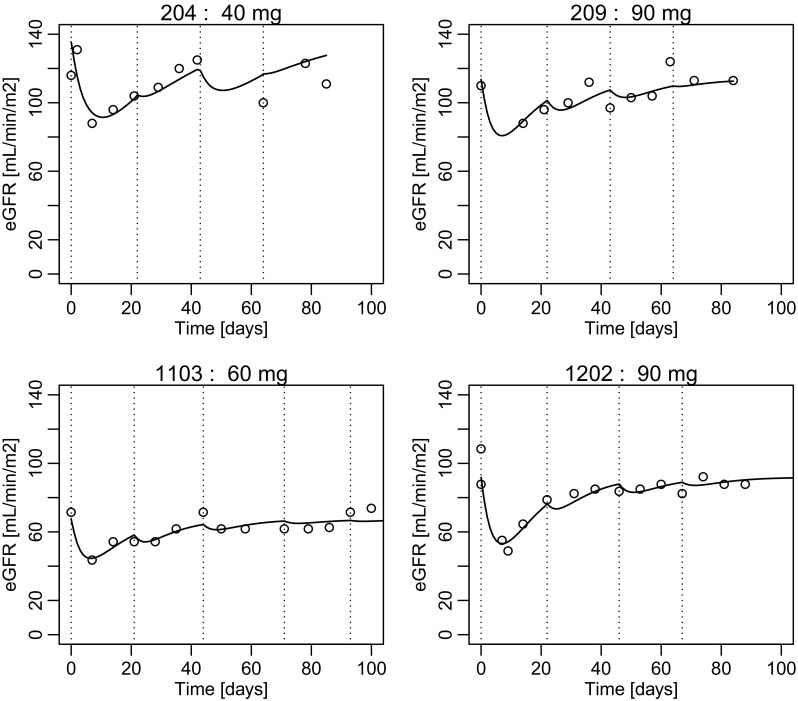
Example of individual time course for the estimated glomerular filtration rate. *Circle* shows the observed value, and *curve* shows the model-predicted time course. *Dotted line* shows the administration of NC-6004 (*dotted lines* administration). *eGFR* estimated glomerular filtration rate

Individual C_f_ was derived from population PK analysis. Most of the PK/PD parameters were assumed to be proportional to exp(η) with η assumed to be normal with a mean of 0. As shown in Fig. 1, the amplitude of the eGFR decrease after starting NC-6004 seemed to reduce with repeated administration. Therefore, *I*_max_ was assumed to decrease through repeated administrations and was expressed as follows:

$${{I}_{\text{max}}}_{\text{ijk}}={{I}_{\text{max}}}_{0}\times \exp \left( -\text{trend}\times {{t}_{j}} \right)\times \exp \left( \eta _{\text{ik}}^{\text{IOV}\left( {{I}_{\text{max}}} \right)} \right),$$

where *i, j*, and k represent the subject, measurement time, and occasion (i.e., the period through *k*th to [*k* + 1]th administration). Inter-occasional variability (IOV) was also assumed because some patients showed the largest eGFR decrease after a later administration rather than after the first administration.

During and after model building, simulations were done. The serum creatinine (sCr) simulation was done by calculating the eGFR using the Cockcroft–Gault equation in both clinical studies, with the increase of sCr ratio from baseline being required to be the same as the decrease in the eGFR ratio within a subject (i.e., an inverse relation within a patient). The mean and standard deviation for the baseline sCr in the NC-6004-003 study (0.772 ± 0.124 mg/dL) were used for the sCr simulation on day 7, which was 6 days after the first administration.

Renal dysfunction severity was calculated for each virtual subject according to the Kidney Disease: Improving Global Outcomes (KDIGO) definition: stage 1 = 0.3 mg/dL increase from or 1.5–1.9 times the pretreatment value; stage 2 = 2.0–2.9 times the pretreatment value; and stage 3 = ≥3.0 times the pretreatment value or the sCr increase >4.0 mg/dL from the pretreatment value [4].

## Results

### Patient characteristics

The patients’ demographics are summarized in Table 1. The mean age of the ten male and two female patients was 59.5 years, ranging from 34 to 72 years. In total, seven patients had carcinoma and five patients had neuroendocrine tumor. Eight patients were classified as stage IV, and four patients were not classified. All patients had metastatic lesions. The most common site of metastasis was the lymph nodes, followed by the liver. All patients had a previous history of chemotherapy, and seven patients had received cisplatin treatment. One patient had undergone a previous gemcitabine treatment. The maximum cumulative dose of cisplatin was 420 mg/m^2^. Six patients had a history of surgery, and three had a history of radiotherapy.

**Table 1:** Patient demographics (safety population)

Characteristics	Cohort		Total (%)
	60 mg/m^2^ (%)	90 mg/m^2^ (%)	
Age (year)			
Number	3	9	12
Mean ± SD	46.3 ± 13.7	63.9 ± 7.5	59.5 ± 11.7
Median	44	66	65
Range	34–61	46–72	34–72
Gender			
Male	2 (66.7)	8 (88.9)	10 (83.3)
Female	1 (33.3)	1 (11.1)	2 (16.7)
Tumor type			
Sarcoma	0 (0.0)	0 (0.0)	0 (0.0)
Carcinoma	1 (33.3)	6 (66.7)	7 (58.3)
Lymphoma	0 (0.0)	0 (0.0)	0 (0.0)
Other	2 (66.7)	3 (33.3)	5 (41.7)
Metastatic sites (multi-choice)			
Adrenal	0 (0.0)	0 (0.0)	0 (0.0)
Liver	3 (100.0)	4 (44.4)	7 (58.3)
Bone	1 (33.3)	0 (0.0)	1 (8.3)
Lymph nodes	3 (100.0)	5 (55.6)	8 (66.7)
Lung	1 (33.3)	2 (22.2)	3 (25.0)
Skin/soft tissue	0 (0.0)	0 (0.0)	0 (0.0)
Other	1 (33.3)	4 (44.4)	5 (41.7)
N/A	0 (0.0)	0 (0.0)	0 (0.0)
Stage			
III	0 (0.0)	0 (0.0)	0 (0.0)
IIIA	0 (0.0)	0 (0.0)	0 (0.0)
IIIB	0 (0.0)	0 (0.0)	0 (0.0)
IV	3 (100.0)	5 (55.6)	8 (66.7)
Previous cancer treatment			
Surgery			
No	2 (66.7)	4 (44.4)	6 (50.0)
Yes	1 (33.3)	5 (55.6)	6 (50.0)
Chemotherapy			
No	0 (0.0)	0 (0.0)	0 (0.0)
Yes	3 (100.0)	9 (100.0)	12 (100.0)
Radiotherapy			
No	2 (66.7)	7 (77.8)	9 (75.0)
Yes	1 (33.3)	2 (22.2)	3 (25.0)
Other therapies			
No	3 (100.0)	8 (88.9)	11 (91.7)
Yes	0 (0.0)	1 (11.1)	1 (8.3)
ECOG performance status			
0	2 (66.7)	5 (55.6)	7 (58.3)
1	1 (33.3)	4 (44.4)	5 (41.7)
2	0 (0.0)	0 (0.0)	0 (0.0)
Hydration			
With	–	5 (55.6)	5 (41.7)
Without	3 (100.0)	4 (44.4)	7 (58.3)

*ECOG* Eastern Cooperative Oncology Group

### Safety analysis

Patients were planned to receive NC-6004 at the doses of 60, 90, 120, and 150 mg/m^2^ but were actually treated with only 60 and 90 mg/m^2^ doses because of the development of the grade 3 or 4 drug-related toxicities, as shown in Table 2. Grade 3 or greater adverse events (AEs) related to NC-6004 included a neutrophil count decrease in the (two patients: 66.7%) 60 mg/m^2^ cohort and (six patients: 66.7%) 90 mg/m^2^ cohort and a decreased white blood cell count in the (four patients: 44.4%) 90 mg/m^2^ cohort. At least one AE related to NC-6004 was observed in all 12 patients in the safety evaluation cohort. Events in which the incidence was more than 40% were neutropenia (10 patients: 83.3%), leukopenia (10 patients: 83.3%), and thrombocytopenia (nine patients: 75.0%).

**Table 2:** Grade 3 or 4 NC-6004-related adverse events

MedDRA SOC	MedDRA PT	60 mg/m^2^, *N* = 3	90 mg/m^2^, *N* = 9
Investigations	Gamma-glutamyltransferase decreased	0 (0.0)	1 (11.1)
Investigations	Neutrophil count decreased	2 (66.7)	6 (66.7)
Investigations	Platelet count decreased	1 (33.3)	1 (11.1)
Investigations	White blood cell count decreased	1 (33.3)	4 (44.4)
Metabolism and nutrition disorders	Hypokalemia	0 (0.0)	1 (11.1)

*MedDRA* Medical Dictionary for Regulatory Activities, *SOC* System Organ Class, *PT* preferred terms

The DLTs in each cohort were counted during the NC-6004 monotherapy phase (cycle 1) and the NC-6004-plus-gemcitabine combination phase (cycle 2). No DLTs occurred in the 60 mg/m^2^ cohort of three patients, but four DLTs were observed in the 90 mg/m^2^ cohort of nine patients. In total, four of 12 patients experienced DLT: three patients experienced grade 4 hematologic toxicities, and one patient experienced a non-hematologic toxicity (an eGFR <30 mL/min/1.73 m^2^). After observing the effect on eGFR, the study protocol was amended to add prophylactic hydration therapy [5] before and after treatment with NC-6004 to prevent renal toxicity. Seven patients did not receive hydration therapy, and other five patients did. No renal toxicity occurred among the patients who received hydration therapy. Based on these findings, the MTD and RD of NC-6004, when given in combination with gemcitabine, were both determined to be 90 mg/m^2^, with the latter being decided after discussion with the study sponsor.

### Efficacy analysis

One patient showed partial response and eight patients had stable disease. The objective response rate and disease control rate were 9.1 and 81.8%, respectively. Patient survival beyond 18 months after the first dose was analyzed by Kaplan–Meier survival curves. The curve reached a 50% survival level by day 327, which persisted until day 596 (i.e., the final follow-up day).

### Population PK analysis

The number of subjects included for PK analysis was 12 from this study and 17 from the UK study, resulting in a total of 546 observed concentrations for the final analysis. The total plasma Pt concentration was measured by the ICP method in all 12 patients in this study (108 points), while in the UK study total plasma Pt concentration was measured by the ICP method in five patients (42 points) and by the AA method in 17 patients (144 points). Notably, in the five patients whose total plasma Pt concentration was measured by both ICP and AA, the AA values were almost 70% higher than the ICP values, so the data obtained by the AA method were converted to ICP values. The plasma-free Pt concentration was also measured in 28 patients (252 points). The selected population PK model was confirmed to explain the NC-6004 PK profile (free concentration also showed a good fit, but the data are not shown).

The PK parameters in the final model are summarized in Table 3. During covariate selection, the influence of ethnic difference on clearance was evaluated to have a ΔOFV of 0.014 (*P* = 0.906). However, the influences of body weight on the clearance and central volume of distribution were significant. Clearance and central volume of distribution increased in proportion to around the 0.7th power for body weight increase, as observed with other drugs. This has also become a good rationale of dose adjustment by body surface area. The typical central volume of distribution was 2.69 L, which was smaller than the blood volume. Therefore, most of the administered NC-6004 was thought to be captured within vessels just after administration. The peripheral volume of distribution was 3.91 L and the amount of NC-6004 transferred to peripheral tissue was considered to be limited. Therefore, this drug should be barely transferable to some vulnerable tissues. Finally, the clearance of NC-6004 was estimated at 0.0687 L h, corresponding to 1/100th of the normal GFR value, meaning that NC-6004 should be stable in the human body.

**Table 3:** Estimated population PK parameters (the final model)

Parameter	Population mean	Inter-individual variability (CV%)
Inter-individual variability		
TV1^a^ (L)	2.69 (2.39–2.99)	13.9 (7.3–18.2)
Θ_(V1−WT)_: V1 = TV1× (WT/67.8) ^Θ^	0.763 (0.412–1.11)	–
V2^†^ (L)	3.91 (2.80–5.02)	–
CL (L/h)	0.0687 (0.0615–0.0759)	17.2 (8.9–22.7)
Θ_(CL−WT)_: CL = TCL× (WT/67.8) ^Θ^	0.731 (0.346–1.115)	–
* Q* (L/h)	0.0117 (0.0096–0.0138)	20.1 (0–28.5)
Vm1 (L)	81.6 (69.7–93.5)	24.4 (0–36.5)
Vm2 (L)	665 (549–781)	42.4 (24.2–54.9)
CLm (L/h)	6.54 (5.56–7.52)	45.7 (21.8–60.9)
Qm (L/h)	9.30 (5.73–12.87)	54.4 (22.2–73.7)
Difference by method [folds] (AA to ICP)	1.69 (1.41–1.97)	21.2 (4.8–29.6)
Intra-individual variability		
Total Pt (ICP) (CV%)	17.0 (15.2–18.7)	
Total Pt (AA) (CV%)	31.6 (27.5–35.3)	
Free Pt (ICP) (CV%]	31.6 (24.0–37.7)	

Mean (90% CI)

*CL* clearance, *V1* central volume of distribution, *V2* volume of distribution, *CV*% coefficient of variation, *ICP* inductively coupled plasma method, *AA* atomic absorption spectroscopy

^a^The typical value at 67.8 kg of body weight

### Population PK/PD analysis

The estimated population PK/PD parameters are shown in Table 4. The number of subjects for the PK/PD analysis was 12 (mean 8.1 points/subject) from this study and 17 (mean 13.7 points/subject) from the UK study (NC-6004-001 study).

**Table 4:** Estimated population pharmacokinetic/pharmacodynamic model parameters

Parameter	Population mean	Inter-individual variance (CV%)
GFR_0_ (mL/min/1.73 m^2^)	83.4	26.5
*K*_out_ (/day)	0.171	48.7
IC_50_ (ng/mL)	35.3	211.7
*I*_max0_	0.851	–
*I*_max_ decrease trend (/day)	0.0273	–
IOV (*I*_max_) (CV%)		53.1
Intra-individual variance (CV%)		8.8

*GFR*_*0*_ baseline GFR; *I*_max_ maximum inhibition (0 < *I*_max_ < 1); *IC*_*50*_ the concentration to achieve 50% inhibition; *GFR*, glomerular filtration rate

**Fig. 2:**
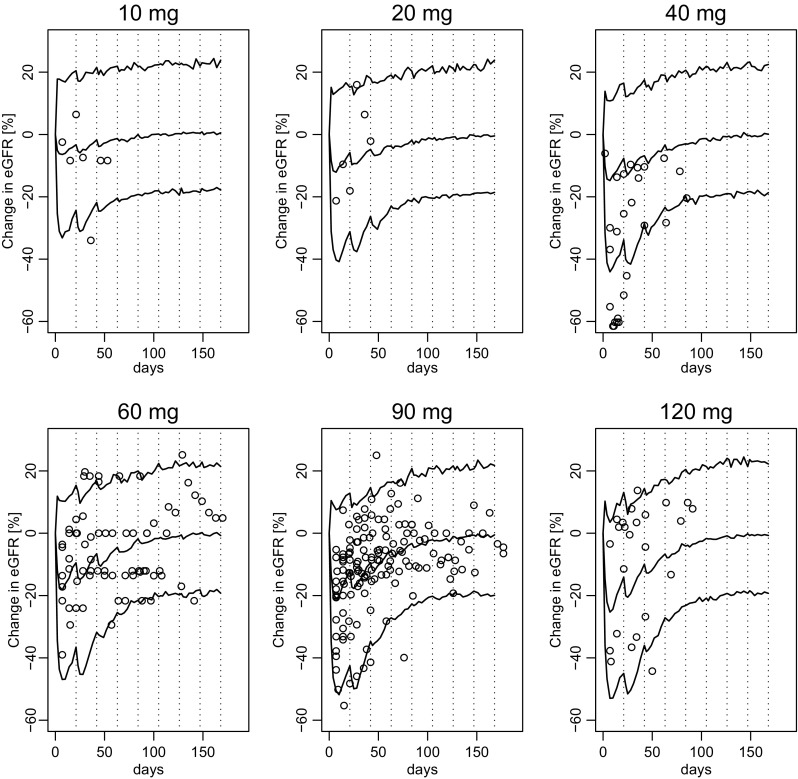
Model prediction showing 80% confidence intervals (CIs) for change in estimated glomerular filtration rate and the observed values. Curves show 90% and 10% quintiles and the median simulated values, with the dotted lines showing the administration timings. Abbreviations: eGFR, estimated glomerular filtration rate

The decrease in eGFR (%) distribution per dose level was simulated and compared with the observed eGFR decrease. As shown in Fig. 2, most observed values were captured within 80% confidence intervals (CIs). The estimated *K*_out_ suggests that the injured kidney function (eGFR) recovered to half the damage level by 4 days and that there was a relatively large difference in this recovery speed among patients [inter-individual coefficient of variation (CV%) of 48%].

The population mean of IC_50_ (i.e., the concentration of free Pt that causes half of the maximum damage) was estimated at 35.3 ng/mL. There was a huge variability in the IC_50_ value, with a CV% of 211.7, meaning that there was a very broad range of susceptibility to free Pt exposure. The IC_50_ and K_out_ values did not change with several sensitive analyses (data not shown).

The value of *I*_max_ decreased with repeated administration of NC-6004, such that the patients became increasingly tolerant with the *I*_max_ decreasing to half by day 25.4. The IOV was assumed in the PK/PD model and remained symmetrical over time (data not shown). The effect of hydration therapy was evaluated preliminarily and was shown to be associated with a ΔOFV decrease by 2.556 (*P* < 0.110). The small number of patients (only five) precluded evaluation of its effect.

The hypothesis of *I*_max_=1 was evaluated preliminarily, and the ΔOVF decreased by 1.77 (*P* < 0.183). However, the IOV for I_max_ was necessary and fixing I_max_ to 1 was thought not to be appropriate. The estimated 90% CI did not include one; however, it was decided that I_max_<1 should remain in the model.

**Fig. 3:**
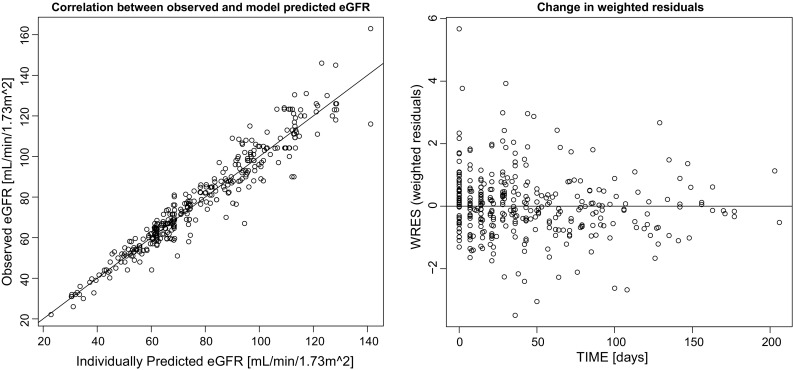
Observed data vs. individual predicted data and weighted residuals vs. time for estimated glomerular filtration rate. *eGFR* estimated glomerular filtration rate, *IPRED* individual predicted data, *DV* observed data, *WRES* weighted residuals

Figure 3 shows the correlation between the individual model prediction for eGFR and the observed eGFR. There was a good correlation, meaning that the model could sufficiently describe the decrease in eGFR after NC-6004 administration. There was no bias or symmetry over time in the weighted residuals (data not shown), so it was concluded that the model described the eGFR changes without bias for the whole period.

### Simulation of provability of acute kidney injury between NC-6004 and cisplatin

The effect of cisplatin on acute kidney injury (AKI) was previously reported in patients with head and neck cancer [4]. All patients had received hydration in that study and the mean cisplatin dose was 99 ± 9 mg/m^2^. According to the KDIGO definition of severity, of the 233 included patients, 158 (68%) developed AKI; among these, 77 (49%) developed stage 1 AKI, 55 (35%) developed stage 2 AKI, and 26 (16%) developed stage 3 AKI.

**Fig. 4:**
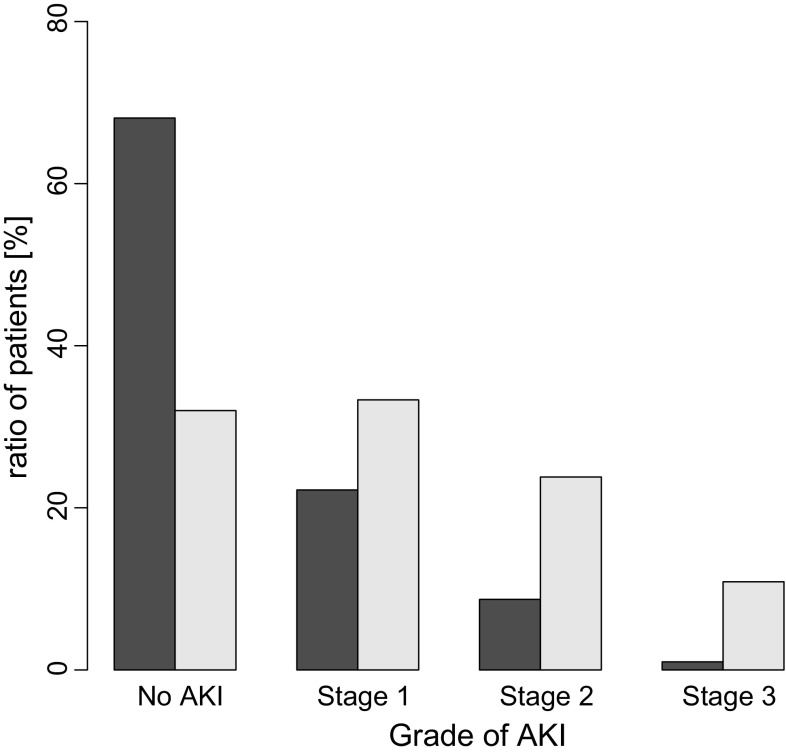
Comparison of acute kidney injury between NC-6004 and cisplatin. Patients (%) are shown by acute kidney injury (AKI) stage (*black bar*) for cisplatin (99 ± 9 mg/m^2^) on days 3–4 [4] and (*gray bar*) as simulated for NC-6004 (100 mg) on day 6

The simulation results for NC-6004 at a dose of 100 mg/m^2^ dose are compared with these results [4] in Fig. 4. Almost 70% of patients who received 100 mg/m^2^ of NC-6004 did not show AKI, and this ratio was double that shown with cisplatin. By comparison, the most common AKI grade was stage 1 with cisplatin treatment, with cisplatin causing a proportionally greater number of cases with AKI as the stage increased, when compared with NC-6004 (there were very few cases with stage 3 AKI). Although the percentage of patients with no AKI became less than 60% at an NC-6004 dose of 200 mg/m^2^, there were still few cases with stage 3 AKI and the overall safety profile did not change substantially from that for the 100 mg/m^2^ dose.

## Discussion

### Safety evaluation

The safety profile of NC-6004 was expected to be similar to that of cisplatin. Therefore, neural, auditory, renal, and hematologic toxicity were considered potential AEs of NC-6004.

Concerning the occurrence of renal toxicity, sCr increased in two patients (16.7%; one given 60 mg/m^2^ and one given 90 mg/m^2^). Renal disorder was also observed in two patients in the 90 mg/m^2^ cohort (16.7%), with renal impairment occurring in one patient in the 90 mg/m^2^ cohort (8.3%). These renal effects were all considered related to NC-6004. But after adding prophylactic hydration therapy before and after treatment with NC-6004, no further renal toxicity events occurred. The detail of the renal toxicity profile was revealed by population PK/PD analysis, which confirmed that the frequency and severity of AKI were much lower than those reported for cisplatin.

There was evidence of hematologic toxicity, with neutropenia being observed in 10 patients (83.3%; three given 60 mg/m^2^ and seven given 90 mg/m^2^), thrombocytopenia in nine patients (75.0%; three given 60 mg/m^2^ and six given 90 mg/m^2^), and leukopenia in 10 patients (83.3%; three given 60 mg/m^2^ and seven given 90 mg/m^2^). Causality was attributed to NC-6004 for all of these events. However, no cases of neutropenia or thrombocytopenia were observed in the NC-6004 monotherapy phase, with both only occurring later the next day after starting gemcitabine. According to its package insert, myelosuppression is the most common AE with gemcitabine [6], and the package insert for cisplatin also states that myelosuppression may occur during treatment [7]. Hematologic toxicities that more than doubled in incidence between cycle one and cycle two and were experienced by at least two patients were anemia, neutropenia, thrombocytopenia, and leukopenia in the 60 mg/m^2^ cohort and hemoglobin decrease, neutropenia, thrombocytopenia, and leukopenia in the 90 mg/m^2^ cohort.

Although a grade 1 peripheral nerve disorder was observed in one patient (8.3%), this neurotoxicity was not considered to be related to NC-6004. Hypersensitivity was also observed in one patient (8.3%), but was judged to be an allergic reaction to the transfusion, and not to NC-6004. In addition, no auditory toxicity was observed. Adverse reactions showed the same pattern as AEs. Finally, six patients (50.0%) from the safety population died within the 18-month follow-up period after the first dose. All of the deaths were considered to be caused by disease progression and were judged to be unrelated to NC-6004.

### Dosing: justification of the MTD and RD

The MTD and RD of NC-6004 were determined to be 90 and 60 mg/m^2^, respectively, based on the protocol criteria. However, the RD was determined to be 90 mg/m^2^ for the following reasons after further discussion with the sponsor: (1) three of the four observed DLTs were considered typical, manageable hematologic toxicities of gemcitabine or cisplatin, and (2) the remaining DLT of cisplatin was shown to be controllable by prophylactic hydration.

#### Population PK and PK/PD analyses

The PK profile of NC-6004 was not influenced by ethnicity, i.e., difference between Japanese and Caucasians with population PK analysis.

The PK/PD model also explained the time course for eGFR after the first dose, as well as the significant decrease in amplification after repeated administrations. The eGFR decrease was thought to reach saturation at 100 mg/m^2^. Finally, although cisplatin had been reported to cause AKI in almost 70% of patients, the PK/PD simulation indicated that NC-6004 caused AKI in only 30% of patients. Most patients showed stage I AKI overall, but while there were high levels of stage II and III AKI with cisplatin, the distribution of AKI shifted in favor of no AKI or milder AKI with NC-6004 therapy.

## Abbreviations

PK: Pharmacokinetic
AKI: Acute kidney injury
PD: Pharmacodynamic
eGFR: Estimated glomerular filtration rate
KDIGO: Kidney disease: improving global outcomes
DLT: Dose-limiting toxicity
Pt: Plasma total platinum
ICP: Inductively coupled plasma

## References

[1] Uchino H, Matsumura Y, Negishi T (2005). Cisplatin incorporating polymeric micelles (NC-6004) can reduce nephrotoxicity and neurotoxicity of cisplatin in rats. Br J Cancer.

[2] Matsumura Y, Maeda H (1986). A new concept for macromolecular therapeutics in cancer chemotherapy: mechanism of tumoritropic accumulation of proteins and the antitumor agent SMANCS. Cancer Res.

[3] Sharma A, Jusko W (1998). Characteristics of indirect pharmacodynamic models and applications to clinical drug responses. Br J Clin Pharmacol.

[4] Bhat ZY, Cadnapaphornchai P, Ginsburg K, Sivagnanam M, Chopra S, Treadway CK (2015). Understanding the risk factors and long-term consequences of cisplatin-associated acute kidney injury: an observational cohort study. PLoS One.

[5] Marcello T (2007). Short hydration regimen and nephrotoxicity of intermediate to high-dose cisplatin-based chemotherapy for outpatient treatment in lung cancer and mesothelioma. Tumori.

[6] Package insert of Gemsar [JAPAN], vol. 15, October 2013

[7] Package insert of Randa [JAPAN], August 2014

